# Systematic review of the pharmacological treatment of alcohol use disorders in individuals infected with hepatitis C

**DOI:** 10.1186/s13722-015-0029-2

**Published:** 2015-02-24

**Authors:** Alexis Thibault, Suzanne Brissette, Didier Jutras-Aswad

**Affiliations:** Research Center, Centre hospitalier de l’Université de Montréal (CRCHUM), 900 St-Denis Street, Montreal, H2X 0A9 QC Canada; Department of Psychiatry, Université de Montréal, Montreal, Canada; Department of Family Medicine, Université de Montréal, Montreal, Canada

**Keywords:** Alcohol, Alcohol dependence, Hepatitis C, Disulfiram, Baclofen, Relapse prevention

## Abstract

Treating alcohol use disorders (AUD) is critical in individuals suffering from hepatitis C infection (HCV). Aside from psychosocial interventions, pharmacological treatment is effective for decreasing alcohol consumption and promoting abstinence. However, unique factors belonging to HCV-infected individuals, such as baseline hepatic vulnerability and possible ongoing hepatitis C treatment, complicate AUD drug therapy. The goal of this review is to systematically identify, summarize, and evaluate the existing evidence on the pharmacological management of AUD in HCV-infected individuals. MEDLINE, Embase, PsycINFO, and the Cochrane Central Register of Controlled Trials were searched for English- and French-language articles published from 1993 to December 2013. The search criteria focused on clinical trials and observational studies assessing the efficacy and/or safety of pharmacological management of AUD in patients infected with HCV. Of 421 identified studies, three were included for analysis. Two were observational studies assessing the safety of disulfiram. One was a randomized controlled trial assessing the efficacy and safety of baclofen. There is paucity of data regarding the efficacy and safety of pharmacological treatment of AUD in HCV-infected individuals, with studies being small series and showing significant heterogeneity. No strong recommendations can be made based on the current studies as to which pharmacological option should be preferred in this sub-population.

## Introduction

Extensive literature has emerged on the dangers of alcohol consumption in patients suffering from hepatitis C virus (HCV) infection. Alcohol use disorders (AUD) are more likely to occur in HCV(+) patients than in the general population [1-3]. In turn, patients with AUD are 3 to 30 times more likely to be HCV(+) compared with the general population (5–50% prevalence) [4], making the comorbid association a frequently encountered clinical challenge. This is of even greater importance given the synergistic interaction of both conditions on the development of liver pathology [5-7]. Approximately 80 percent of all HCV-infected individuals will progress to chronic HCV infection. Of these, 10–15 percent will advance to cirrhosis within the first 20 years. Cirrhotic individuals are, in turn, at increased risk of developing hepatocellular carcinoma [8]. Patients with chronic HCV infection and superimposed AUD develop accelerated fibrosis, as well as higher rates of cirrhosis, liver failure, and hepatocellular carcinoma, compared to HCV(+) patients who do not drink or to HCV(-) drinkers [4,9]. Moreover, alcohol consumption reduces the effectiveness of interferon-based therapies [4,9,10]. This may be explained by biological mechanisms, such as increased viral resistance [11] and altered immunity [12], but also by lower compliance and higher dropout rates in patients with AUD [13,14]. This, combined with the psychiatric effects of HCV treatment, predisposes patients to continued drinking and perpetuates the cycle of AUD and liver injury [15].

Major associations, such as the European Association for the Study of the Liver and the American Association for the Study of Liver Diseases, have stressed the need to address alcohol use in infected patients. While aiming for complete abstinence is desirable, reduction of drinking may be an acceptable alternative goal [16-18]. The Centers for Disease Control and Prevention has recently recommended to systematically screen HCV(+) patients for alcohol use and to treat problem drinkers accordingly [19]. However, no direct recommendation regarding the preferred treatment options for AUD in HCV(+) patients has been issued, except for brief counseling and referral to appropriate care [19].

In the general population, psychotherapeutic approaches for AUD play an important role in reducing alcohol use and attaining abstinence. However, when given alone, such interventions are not always successful, and relapse rates after long-term treatment exceed 40–70 percent [20-23]; available medications to treat AUD are thus critical in relapse prevention strategies [24,25]. Research evidence demonstrates that physicians without extensive specialized training can effectively prescribe such medications, leading to a reduction in heavy alcohol drinking and increased days of abstinence [26].

However, from a clinical point of view, unique factors belonging to the patients infected with HCV complicate AUD drug therapy, making this population different from the HCV(-) drinkers. Aside from the existing epidemiological and etiological association described above, the baseline hepatic vulnerability and possible additional hepatotoxicity caused by certain compounds used to treat AUD make their utilization in HCV(+) drinkers potentially more challenging than in HCV(-) ones. The natural history of chronic HCV infection, known to cause waxing and waning of liver enzyme elevations over time, can confound medication studies, especially safety monitoring of a therapeutic challenge in a given subject [27]. Specific issues stem from possible interactions between the drugs used to treat AUD and the ones used to treat HCV infection, both at a pharmacological and psychiatric level. We thus propose in this article to systematically review the evidence on the pharmacological management of AUD in individuals infected with HCV.

## Review

### Methods

#### Search strategy

The literature search was conducted in the following electronic databases: MEDLINE, Embase, PsycINFO, and the Cochrane Central Register of Controlled Trials. The search was restricted to English- and French-language articles published from 1993 to December 2013 (see Table 1 for the detailed search strategy). Additional search was conducted on clinicaltrials.gov (included in Table 1) to identify undergoing, unpublished trials. The search strategy was elaborated with the input of a medical library specialist (see acknowledgments). The methodological aspect of this article is based on Grimshaw’s “A Knowledge Synthesis Factor,” available online at the Canadian Institutes of Health Research website [28]. Titles and abstracts were sorted and two MD-level reviewers (AT and SB) independently evaluated potentially relevant studies. Additionally, related articles and their references were reviewed manually to identify additional eligible studies. A third researcher (DJA) was available if any discrepancy occurred between the searches led by the reviewers.

**Table 1:** **Search strategy to identify studies, by database**

**Database**	**Search terms**	**Number of hits**	**Number of included articles**
MEDLINE (Search terms via Pubmed)*	(“Hepatitis C” OR HCV) AND (“alcohol dependence” OR alcoholism OR “alcohol-dependent”) AND (pharmacological treatment OR drug therapy OR disulfiram OR naltrexone OR acamprosate OR baclofen OR ondansetron OR topiramate)	138	3
Embase (MeSH terms via Ovid)	#1: Hepatitis C OR Hepatitis C virus	92110	3 (duplicates)
	#2: Alcoholism [subheading: drug therapy]	5267	
	#3: Disulfiram	7571	
	#4: Naltrexone	11449	
	#5: Acamprosate	1772	
	#6: Baclofen	14259	
	#7: Topiramate	14850	
	#8: Ondansetron	12818	
	#9: #2 OR #3 OR #4 OR #5 OR #6 OR #7 OR #8	60923	
	#10: #1 AND #9	237	
	#11: Limit #10 to human studies and year 1993 to December 2013 (final result)	225	
PsycINFO (MeSH terms via Ovid)	#1: Hepatitis	1821	2 (duplicates)
	#2: Alcoholism	24628	
	#3: #1 AND #2	62	
	#4: Limit #3 to human studies and year 1993 to December 2013	54	
Cochrane Central Register of Controlled Trials (MeSH terms via Ovid)	#1: Alcoholism	2130	0
	#2: Hepatitis C	707	
	#3: #1 AND #2	4	
	#4: Limit #3 to year 1993 to December 2013	4	
Clinicaltrials.gov (unpublished)	“Hepatitis C” AND “alcohol”	149	1 (underway; no results yet)

*limits: human studies and studies between 1993 and December 2013.

#### Eligibility criteria

To be included, studies had to evaluate the efficacy or safety of drug therapy for AUD in participants infected with HCV. Given the paucity of data and to avoid the exclusion of studies with relevant but less-than-optimal data, we chose broad inclusion criteria.

All types of studies were included: randomized controlled trials (RCTs); observational, retrospective, and prospective studies; and case reports. Post-hoc analysis of primary studies and studies where efficacy and safety of a given drug were not the primary measured outcomes were also included. Reviews were excluded.

Participants were either inpatients or outpatients, 18 years of age or older, diagnosed with HCV infection at any stage of the disease, with concomitant AUD.

#### Data extraction and analysis

When available, the following data were retrieved from the included studies: authors, publication year, study design, length of the study, inclusion and exclusion criteria, sample size, characteristics of participants, type of intervention, measured outcomes, follow-up rate, and compliance.

## Results

**Figure 1:**
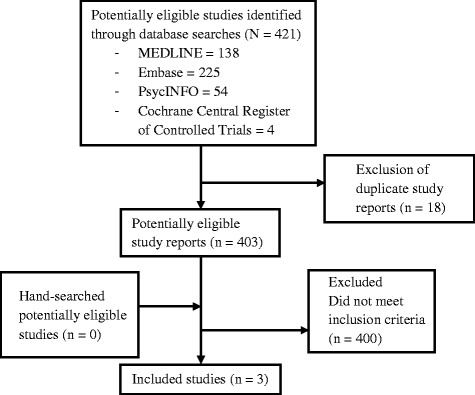
**Flow chart of the selection process of published studies.**

We identified 421 potentially eligible studies. Of these, 418 were excluded after reviewing titles and/or abstracts because they had no relevance to the subject of this review or because they were duplicates. No studies were excluded because of study design or participant characteristics (see Figure 1 for selection process). Out of the three selected studies, two were observational studies on disulfiram therapy and one was an RCT on baclofen. Neither of the three studies reported data on participants with acute HCV infection (see Table 2 for detailed search results).

**Table 2:** **Included published studies assessing the pharmacological management of AUD in participants infected with HCV**

**References**	**Study type and duration**	**Participants**	**Intervention**	**Measured outcomes**	**Results**	**Adverse events**
Martin et al. [29]	Retrospective controlled study, 12 months	26 HCV(+) men versus 20 HCV(-) (19 men, 1 woman)	Disulfiram 1500 mg/week, supervised	Hepatic safety of disulfiram as measured by AST/ALT	1–No statistically or clinically significant elevations of AST/ALT for the HCV(+) group at any time point	No patients taken off disulfiram; no subjects reached 3 times to upper limit for AST/ALT
				1–In HCV(+) group over time; 2–Compared to HCV(-) group	2–Between-group means were identical at all time points	
Saxon et al. [27]	Prospective study of 3 months	57 male veterans; 18 HCV(+) and 39 HCV(-) with elevation of AST/ALT	Disulfiram	Hepatic safety of disulfiram as measured by elevations in AST/ALT in	Clinically relevant elevation (2x the baseline and 3x the normal) in:	No hepatitis or hepatic failure, although some patients did have a significant elevation
				1–HCV(+)	1–4/18 HCV(+)	
				2–HCV(-) with baseline elevation of AST/ALT	2–1/39 HCV(-)	
Leggio et al. [31]	Post-hoc analysis of a randomized placebo-controlled trial of cirrhotic patients [30], 12 weeks	24 HCV(+) cirrhotic patients; 12 receiving baclofen versus 12 receiving placebo	Baclofen 30 mg daily versus placebo	1–Efficacy of baclofen in HCV(+) cirrhotic patients	1–Significantly more abstinence in baclofen group (83,3% vs. 25%; p = 0,0123)	No hepatic or renal adverse events. General side effects were not more frequent compared to placebo
				2–Hepatic safety of baclofen as measured by biochemical liver function tests	2–Higher albumin and trend toward decreased INR in baclofen group; no effect on AST/ALT/GGT	

AST: aspartate transaminase, ALT: alanine transaminase GGT: gamma-glutamyl transferase, INR: international normalized ratio.

The separate search on clinicaltrials.gov yielded 149 results, one of which (NCT01008280) was of interest and fitted our inclusion criteria: an ongoing RCT assessing the efficacy of baclofen. It was not formally included in our analysis for there are no published results yet (not included in Table 2 or Figure 1).

### Included studies

The use of disulfiram in HCV(+) individuals with AUD was first assessed by Saxon et al. in a study focusing on this medication’s safety [27]. They reported a prospective study of 57 males diagnosed with alcohol dependence (according to DSM-IV criteria) and concomitant elevated liver function test results and/or serologic evidence of HCV infection. All participants received disulfiram. Eighteen out of the 57 subjects were HCV(+). Sequential liver function tests were conducted at baseline and for up to 3 months while patients were taking the medication. The efficacy of the drug was not a measured outcome. The presence of aspartate transaminase (AST) or alanine transaminase (ALT) values above 200 u/l led to the exclusion of two subjects as determined in the protocol. Of the 18 HCV(+) participants, four exhibited marked transaminase elevations during the trial, as defined by transaminase levels greater than twice the baseline and three times the normal range. In comparison, only 1 out of 39 subjects without HCV had a marked increase of those enzymes. Although this comparison was not statistically evaluated, one of the conclusions of the report was that under disulfiram treatment, HCV(+) alcohol-dependent participants were more likely to have significant transaminase elevations compared to HCV(-) participants with abnormal baseline liver enzymes. The authors also stated that their findings support the cautious use of this medication with regular laboratory monitoring in patients who present with elevated liver enzymes at baseline [27].

A second safety study [29] retrospectively assessed transaminase levels in 26 HCV(+) and 20 HCV(-) subjects receiving 1500 mg of weekly supervised disulfiram for 12 months (disulfiram was given in doses either of 500 mg three times weekly or of 750 mg two times weekly). Groups were similar regarding age, ethnicity, and baseline mean AST and ALT levels. Both groups also had similar compliance rates (around 70%) with disulfiram. Within-group analysis revealed no statistically or clinically significant increase (defined by three times the upper normal limit) across time in mean AST and ALT levels at 3, 6, 9, and 12 months for the HCV(+) group. However, AST levels in HCV(-) subjects statistically dropped at 6 and 12 months (p < 0.05). Between-group comparison, using a nonpaired mean, also failed to identify a statistical difference in liver enzyme levels at any time point or in changes across time between HCV(+) and HCV(-) participants. The authors concluded that HCV(+) participants were not more prone to disulfiram-induced hepatotoxicity when compared with HCV(-) subjects. Overall, the report also concluded that supervised disulfiram may be used in HCV(+) AUD patients, with close monitoring of liver enzymes. Of interest, even though no clinically relevant elevations of liver enzymes or need for disulfiram discontinuation occurred, three out of 26 HCV(+) individuals reached two times the upper normal limit, as opposed to none in the HCV(-) group. Moreover, 14 and 16, respectively, out of the 26 HCV(+) participants, experienced AST and ALT elevations compared to their baseline values. However, these results were not statistically different from the ones in the HCV(-) group.

More recently, Addolorato et al. reported reported a study of 84 subjects diagnosed with alcohol dependence according to DSM-IV, with comorbid diagnosis of viral and/or alcoholic cirrhosis [30]. A post-hoc analysis of a subsample of 24 participants who were also suffering from chronic HCV infection was then undertaken by Leggio et al. [31]. HCV(+) alcohol-dependent cirrhotic patients were randomized in two groups; 12 received 30 mg/day of baclofen and the other 12 received placebo. There were no statistical differences in baseline characteristics, including on alcohol dependence’s duration and severity. Regarding the primary outcome, subjects in the baclofen group achieved higher rates of abstinence compared to placebo group (83.3% vs. 25.0%; p = 0.0123). The baclofen group also showed a greater increase of albumin values from baseline (p = 0.0132) and a trend toward decrease in international normalized ratio (INR) levels that did not reach significance (p = 0.0716). No difference was observed in AST, ALT, gamma-glutamyl transferase (GGT), and creatinine values. Dropout rates and side effects were comparable; no participant discontinued the drug for safety reasons. The subsample of 24 HCV(+) alcohol-dependent subjects had comparable baseline characteristics to the initial sample (84 alcohol-dependent participants), except for higher initial ALT and AST levels. Overall, the authors concluded that baclofen was more effective than placebo in the achievement and maintenance of alcohol abstinence, and in the improvement of some liver function tests in alcohol-dependent HCV(+) cirrhotic patients.

A fourth study (NCT01008280 on clinicaltrial.gov) evaluating the efficacy and safety of 12 weeks of 30 mg daily of baclofen in HCV(+) subjects with AUD is presently underway. The U.S. multisite, double-blind, randomized placebo-controlled trial was expected to enroll 180 veterans and be completed in June 2014.

## Discussion

A critical analysis of the identified trials underlines a number of interesting findings and issues that remain unresolved. The two included observational studies of 57 and 46 participants, respectively, assessed the safety of disulfiram in HCV(+) individuals. Both study groups concluded that disulfiram could be a safe therapeutic option, although frequent laboratory monitoring is warranted because of possible hepatotoxicity [27,29]. The third study was a 24-HCV(+) patient, randomized, placebo-controlled trial, which concluded that baclofen was effective in maintaining abstinence. The drug seemed safe in this population, since no elevation of biochemical markers of liver injury was observed. The results even showed improvements in biological markers, when compared to placebo [31].

A number of limitations can be identified in the first disulfiram study [27]. It was not placebo controlled, nor was there a control group comprised of subjects with normal liver function. Also, there was no statistical comparison of the biological markers of disulfiram-induced hepatic injury between HCV(+) and HCV(-) subjects, which may possibly be explained by the lack of power related to the limited number of subjects in this study. Moreover, although baseline values of hepatic function tests were provided for the entire sample, those values were not specifically given for HCV(+) individuals. Other limitations include the absence of information regarding dose and compliance to disulfiram, and lack of assessment of alcohol intake by participants. Consequently, the drawing of strong conclusions regarding disulfiram’s safety is difficult. Both the cause and significance of the enzyme elevations in HCV(+) subjects are not easy to interpret in the study. Such elevations could be related to alcohol consumption, hepatitis C per se, or other characteristics not measured and controlled at baseline. The study is still informative when considering that a relatively small portion of subjects with an HCV(+) status and/or elevated baseline transaminases developed marked liver enzymes elevations while taking disulfiram, and none developed severe adverse events.

In the second study [29], the retrospective design precludes the drawing of definitive conclusions and questions the significance of enzyme elevations. More than half of the data were missing for the 6- to 12- month period. No exclusion criteria were given and no method to assess alcohol intake was applied. On the other hand, the presence of a control group, the HCV(-) AUD participants, with similar baseline characteristics and compliance rates, allows for more reliable conclusion regarding the safety of disulfiram in HCV(+) patients suffering from AUD. The absence of severe transaminase elevations and lack of adverse events is reassuring, as is the absence of discontinuation because of hepatotoxicity.

Some common limitations can be identified in both disulfiram studies. Small sample sizes often preclude observation of rare but potentially serious adverse events. The sole use of AST and ALT as an accurate indicator of hepatic injury may be criticized [32]. However, the cut-off of three times the upper limit of normal has an experimental and clinical basis [33]. The absence of randomization, placebo control, and lack of information regarding effectiveness are also limitations, but both studies remain highly useful as the only ones to have addressed the clinical use of disulfiram therapy in individuals infected with HCV.

The trial assessing baclofen [31] also contains limitations, including the small sample size and those inherent to post-hoc analysis. It would have been interesting to compare the final liver function tests of the HCV(+) subsample to those of the original sample. This would have allowed the comparison of drug safety and effectiveness between HCV(+) cirrhotic subjects and HCV(-) cirrhotic participants. Another limitation is the fact that the results of this study primarily apply to cirrhotic patients. However, safety of baclofen with these patients is reassuring for individuals with less severe liver disease, and conclusions about baclofen’s safety can probably be inferred to all HCV(+) patients. These limitations taken into account, this study seems to provide safety and preliminary efficacy data on baclofen in alcohol-dependent cirrhotic patients suffering from HCV infection. It is the only identified RCT assessing pharmacological management in this population, and the only one using various types of liver function tests, providing a more accurate estimate of hepatic integrity.

Unfortunately, in light of our search, no other studies specifically assessed AUD treatment options in a particular sample of HCV-infected individuals. Some authors agree that the risk of disulfiram liver injury appears much lower than the one caused by excessive alcohol consumption with concurrent HCV infection [10,29]. However, its known limited efficacy in the general population [34-38] and the above-mentioned limitations in the two trials prevent any recommendation regarding disulfiram’s place in a treatment algorithm. Baclofen is the only molecule that has been assessed in an RCT of HCV(+) individuals, and a recent systematic review [39] concluded that the drug could be considered as the preferred option in patients with moderate to severe liver disease in whom other pharmacological treatments are not safe. However, only a few studies have assessed baclofen in the general population, with encouraging yet mixed results [30,40,41], which further limits the sole above-mentioned study [31]. The registered clinical trial assessing baclofen in HCV-infected veterans (NCT01008280) will perhaps answer a few more pending questions.

Several other studies related to the object of this review but not fitting our search criteria merit mention. One randomized placebo-controlled trial [42] and its associated open-label extension phase [43] both reported promising results regarding extended-release naltrexone’s hepatic safety in the treatment of opioid dependence in a sample of adults, which turned out to have high rates (over 80%) of mild to moderate chronic HCV infection. Although HCV(+) subjects were not specifically studied, it is worth noting that no serious adverse events were recorded. A longitudinal cohort study [44] assessing the safety of oral naltrexone given for opioid and/or AUD and its impact on antiretroviral effectiveness in an HIV population has also been conducted. The authors reported very few enzyme elevations during follow-up and concluded that oral naltrexone was a safe option. While this was not the focus of the study, a strong proportion of the sample (57%) was co-infected with HCV. Oral naltrexone is known for its hepatotoxicity but may merit further assessment as to its role in the treatment of subjects with pre-existent and ongoing liver damage. The same can be said about extended-release naltrexone, especially since there is no clear evidence for its hepatoxicity [45,46].

The present systematic review has some significant limitations, including the lack of mechanism to exclude publication bias, as no search for unpublished studies was undertaken other than on clinicaltrials.gov. The small sample size, heterogeneous groups of patients, and variability of study design make the comparison of studies quite limited, and a meta-analysis was impossible to realize. Also, non-English and non-French studies were not included.

The lack of trials may be explained partly by the obstacles in treatment accessibility in AUD patients infected with HCV. Substance-addicted HCV(+) patients have known inherent characteristics such as concomitant mental illness, disorganized lifestyle, and poor socioeconomic status, which greatly limit the accessibility and success of adequate treatment [9]. Additionally, these patients also face barriers at the provider level and system level [47]. Until recently, the inclusion of such patients in studies was even more limited, and often not encouraged. This seems to be somewhat paradoxical since they represent a substantial proportion of patients infected with HCV. Moreover, treating HCV and AUD concomitantly is a relatively recent practice. Thus, the question of the management of such patients is a new one, explaining perhaps the absence of studies examining this specific situation.

The advent of novel antiviral treatments for chronic hepatitis C makes shorter and interferon-free regimens possible. The combinations of direct-acting antiviral agents have fewer psychiatric side effects compared to previous treatment options. Concerns regarding interactions between alcohol and HCV treatment may thus seem of a lesser importance. However, interferon-free regimens will not be available or suit every patient. Moreover, even with the availability of these highly effective therapies, few patients actually receive treatment, multiple barriers and low rates of eligibility still being the norm [48-50]. Heavy alcohol consumption is constantly reported, even with newer treatment regimens, as one of the principal barrier to HCV treatment and is considered a relative contraindication to antiviral treatments [49,51]. Therefore, treating AUD remains an important goal to pursue to maximize availability to newer HCV infection treatment options.

Additional studies assessing the pharmacological management of AUD in HCV(+) individuals are needed, particularly high-quality RCTs evaluating already-studied molecules and other options, such as acamprosate. The latter molecule could be an interesting research target, given its known efficacy and hepatic safety [52]. Nalmefene, gabapentin, and topiramate, all promising therapeutic options for alcohol dependence (but not FDA-approved for this purpose), could also be the focus of future research. Nalmefene, a drug acting on the opioid system, is not known to cause liver injury in the several studies to date [53]. Gabapentin, an antiepilectic, has an extrahepatic metabolism and is considered to have no adverse events on the hepatic system [54]. Topiramate, also an antiepileptic, rarely causes liver enzyme elevations and is considered safe, at least in dosage used for epilepsy. Studies examining the pharmacological management of AUD with concomitant antiviral therapy would be interesting, considering the potential combined effect of both treatment regimens on hepatic integrity and mental health, and potential drug-drug interactions. There is also a need for qualitative and quantitative studies to evaluate the benefits of an integrated approach in the treatment of comorbid hepatitis C and AUD. Such approaches, combining mental health care and medical care, are usually beneficial in the management of such difficult-to-treat populations with similar comorbidities, such as SUD and hepatitis [55-57].

## Conclusion

Co-occurrence of HCV infection and AUD is frequently encountered, and patients afflicted by both conditions are at increased risk of liver disease and subsequent mortality. There is, unfortunately, a small number of studies that examine pharmacological options for AUD treatment in HCV patients. Baclofen specifically showed promising results, while disulfiram’s results, though encouraging, are greatly limited by study designs. Due to the paucity of data, no clinical recommendations regarding pharmacological interventions can be made. The present review and its conclusion stress the need for future research, including studies to examine the efficacy of other existing pharmacological options for AUD in HCV(+) individuals.

## Abbreviations

ALT: Alanine transaminase
AST: Aspartate transaminase
AUD: Alcohol use disorders
GGT: Gamma-glutamyl transferase
HCV: Hepatitis C virus
HCV (+): Hepatitis C virus positive
HCV (-): Hepatitis C virus negative
INR: International normalized ratio
RCT: Randomized controlled trial
SUD: Substance use disorder

## References

[1] Wiley TE, McCarthy M, Breidi L, McCarthy M, Layden TJ (1998). Impact of alcohol on the histological and clinical progression of hepatitis C infection. Hepatology.

[2] Armstrong GL, Wasley A, Simard EP, McQuillan GM, Kuhnert WL, Alter MJ (2006). The prevalence of hepatitis C virus infection in the United States, 1999 through 2002. Ann Intern Med.

[3] Singal AK, Kuo YF, Anand BS (2012). Hepatitis C virus infection in alcoholic hepatitis: prevalence patterns and impact on in-hospital mortality. Eur J Gastroenterol Hepatol.

[4] Singal AK, Anand BS (2007). Mechanisms of synergy between alcohol and hepatitis C virus. J Clin Gastroenterol.

[5] Corrao G, Arico S (1998). Independent and combined action of hepatitis C virus infection and alcohol consumption on the risk of symptomatic liver cirrhosis. Hepatology.

[6] Corrao G, Torchio P, Zambon A, Ferrari P, Arico S, di Orio F (1998). Exploring the combined action of lifetime alcohol intake and chronic hepatotropic virus infections on the risk of symptomatic liver cirrhosis. Collaborative groups for the study of liver diseases in Italy. Eur J Epidemiol.

[7] Harris DR, Gonin R, Alter HJ, Wright EC, Buskell ZJ, Hollinger FB (2001). The relationship of acute transfusion-associated hepatitis to the development of cirrhosis in the presence of alcohol abuse. Ann Intern Med.

[8] Chen SL, Morgan TR (2006). The natural history of hepatitis C virus (HCV) infection. Int J Med Sci.

[9] Cooper CL (2008). Obstacles to successful HCV treatment in substance addicted patients. J Addict Dis.

[10] Kulig CC, Beresford TP (2005). Hepatitis C in alcohol dependence: drinking versus disulfiram. J Addict Dis.

[11] Takahashi K, Takahashi T, Takahashi S, Watanabe K, Boku S, Matsui S (2001). Difference in quasispecies of the hypervariable region 1 of hepatitis C virus between alcoholic and non-alcoholic patients. J Gastroenterol Hepatol.

[12] Szabo G, Aloman C, Polyak SJ, Weinman SA, Wands J, Zakhari S (2006). Hepatitis C infection and alcohol use: a dangerous mix for the liver and antiviral immunity. Alcohol Clin Exp Res.

[13] Grando-Lemaire V, Goisset P, Sorge F, Trinchet JC, Castera L, Roulot D (2002). Hepatitis C virus screening in drug users in an addiction out-patient unit. Gastroenterol Clin Biol.

[14] Anand BS, Currie S, Dieperink E, Bini EJ, Shen H, Ho SB (2006). Alcohol use and treatment of hepatitis C virus: results of a national multicenter study. Gastroenterology.

[15] Zdilar D, Franco-Bronson K, Buchler N, Locala JA, Younossi ZM (2000). Hepatitis C, interferon alfa, and depression. Hepatology.

[16] Ghany MG, Strader DB, Thomas DL, Seeff LB (2009). Diagnosis, management, and treatment of hepatitis C: an update. Hepatology.

[17] Ghany MG, Nelson DR, Strader DB, Thomas DL, Seeff LB (2011). An update on treatment of genotype 1 chronic hepatitis C virus infection: 2011 practice guideline by the american association for the study of liver diseases. Hepatology.

[18] EASL (2011). European association of the study of the liver hepatitis C virus clinical practice guidelines. Liver Int.

[19] Smith BD, Morgan RL, Beckett GA, Falck-Ytter Y, Holtzman D, Ward JW (2012). Hepatitis C virus testing of persons born during 1945–1965: recommendations from the Centers for Disease Control and Prevention. Ann Intern Med.

[20] Finney JW, Hahn AC, Moos RH (1996). The effectiveness of inpatient and outpatient treatment for alcohol abuse: the need to focus on mediators and moderators of setting effects. Addiction.

[21] Anton RF, O’Malley SS, Ciraulo DA, Cisler RA, Couper D, Donovan DM (2006). Combined pharmacotherapies and behavioral interventions for alcohol dependence: the COMBINE study: a randomized controlled trial. JAMA.

[22] Witkiewitz K, Marlatt GA (2007). Modeling the complexity of post-treatment drinking: it’s a rocky road to relapse. Clin Psychol Rev.

[23] Wolwer W, Frommann N, Janner M, Franke PE, Scherbaum N, Lieb B (2011). The effects of combined acamprosate and integrative behaviour therapy in the outpatient treatment of alcohol dependence: a randomized controlled trial. Drug Alcohol Depend.

[24] Pettinati HM, Weiss RD, Miller WR, Donovan D, Ernst DB, Rounsaville BJ (2004). Medical Management Treatment Manual: A Clinical Research Guide for Medically Trained Clinicians Providing Pharmacotherapy as Part of the Treatment for Alcohol Dependence.

[25] Weiss RD, O’Malley SS, Hosking JD, Locastro JS, Swift R (2008). Do patients with alcohol dependence respond to placebo? Results from the COMBINE study. J Stud Alcohol Drugs.

[26] Ernst DB, Pettinati HM, Weiss RD, Donovan DM, Longabaugh R (2008). An intervention for treating alcohol dependence: relating elements of medical management to patient outcomes with implications for primary care. Ann Fam Med.

[27] Saxon AJ, Sloan KL, Reoux J, Haver VM (1998). Disulfiram use in patients with abnormal liver function test results. J Clin Psychiatry.

[28] Grimshaw J. A Knowledge Synthesis Chapter. Ottawa: Canadian Institutes of Health Research. http://www.cihr-irsc.gc.ca/e/documents/knowledge_synthesis_chapter_e.pdf.

[29] Martin B, Alfers J, Kulig C, Clapp L, Bialkowski D, Bridgeford D (2004). Disulfiram therapy in patients with hepatitis C: a 12-month, controlled, follow-up study. J Stud Alcohol.

[30] Addolorato G, Leggio L, Ferrulli A, Cardone S, Vonghia L, Mirijello A (2007). Effectiveness and safety of baclofen for maintenance of alcohol abstinence in alcohol-dependent patients with liver cirrhosis: randomised, double-blind controlled study. Lancet.

[31] Leggio L, Ferrulli A, Zambon A, Caputo F, Kenna GA, Swift RM (2012). Baclofen promotes alcohol abstinence in alcohol dependent cirrhotic patients with hepatitis C virus (HCV) infection. Addict Behav.

[32] Kaplowitz N (2001). Drug-induced liver disorders: implications for drug development and regulation. Drug Saf.

[33] Craxi A, Almasio P (1996). Diagnostic approach to liver enzyme elevation. J Hepatol.

[34] Jorgensen CH, Pedersen B, Tonnesen H (2011). The efficacy of disulfiram for the treatment of alcohol use disorder. Alcohol Clin Exp Res.

[35] Hughes JC, Cook CC (1997). The efficacy of disulfiram: a review of outcome studies. Addiction.

[36] Garbutt JC, West SL, Carey TS, Lohr KN, Crews FT (1999). Pharmacological treatment of alcohol dependence: a review of the evidence. JAMA.

[37] Suh JJ, Pettinati HM, Kampman KM, O’Brien CP (2006). The status of disulfiram: a half of a century later. J Clin Psychopharmacol.

[38] Miller PM, Book SW, Stewart SH (2011). Medical treatment of alcohol dependence: a systematic review. Int J Psychiatry Med.

[39] Muzyk AJ, Rivelli SK, Gagliardi JP (2012). Defining the role of baclofen for the treatment of alcohol dependence: a systematic review of the evidence. CNS Drugs.

[40] Garbutt JC, Kampov-Polevoy AB, Gallop R, Kalka-Juhl L, Flannery BA (2010). Efficacy and safety of baclofen for alcohol dependence: a randomized, double-blind, placebo-controlled trial. Alcohol Clin Exp Res.

[41] Addolorato G, Caputo F, Capristo E, Domenicali M, Bernardi M, Janiri L (2002). Baclofen efficacy in reducing alcohol craving and intake: a preliminary double-blind randomized controlled study. Alcohol Alcohol.

[42] Mitchell MC, Memisoglu A, Silverman BL (2012). Hepatic safety of injectable extended-release naltrexone in patients with chronic hepatitis C and HIV infection. J Stud Alcohol Drugs.

[43] Krupitsky E, Nunes EV, Ling W, Gastfriend DR, Memisoglu A, Silverman BL (2013). Injectable extended-release naltrexone (XR-NTX) for opioid dependence: long-term safety and effectiveness. Addiction.

[44] Tetrault JM, Tate JP, McGinnis KA, Goulet JL, Sullivan LE, Bryant K (2012). Hepatic safety and antiretroviral effectiveness in HIV-infected patients receiving naltrexone. Alcohol Clin Exp Res.

[45] Garbutt JC, Kranzler HR, O’Malley SS, Gastfriend DR, Pettinati HM, Silverman BL (2005). Efficacy and tolerability of long-acting injectable naltrexone for alcohol dependence: a randomized controlled trial. JAMA.

[46] Lucey MR, Silverman BL, Illeperuma A, O’Brien CP (2008). Hepatic safety of once-monthly injectable extended-release naltrexone administered to actively drinking alcoholics. Alcohol Clin Exp Res.

[47] Bruggmann P (2012). Accessing hepatitis C patients who are difficult to reach: it is time to overcome barriers. J Viral Hepat.

[48] McGowan CE, Monis A, Bacon BR, Mallolas J, Goncales FL, Goulis I (2013). A global view of hepatitis C: physician knowledge, opinions, and perceived barriers to care. Hepatology.

[49] Maier MM, He H, Schafer SD, Ward TT, Zaman A (2014). Hepatitis C treatment eligibility among HIV-hepatitis C virus coinfected patients in Oregon: a population-based sample. AIDS Care.

[50] Nunes D, Saitz R, Libman H, Cheng DM, Vidaver J, Samet JH (2006). Barriers to treatment of hepatitis C in HIV/HCV-coinfected adults with alcohol problems. Alcohol Clin nd Exp Res.

[51] Butt AA, Khan UA, Shaikh OS, McMahon D, Dorey-Stein Z, Tsevat J (2009). Rates of HCV treatment eligibility among HCV-monoinfected and HCV/HIV-coinfected patients in tertiary care referral centers. HIV Clin Trials.

[52] Rosner S, Hackl-Herrwerth A, Leucht S, Lehert P, Vecchi S, Soyka M (2010). Acamprosate for alcohol dependence. Cochrane Database Syst Rev.

[53] Soyka M (2014). Nalmefene for the treatment of alcohol dependence: a current update. Int J Neuropsychopharmacol.

[54] Ahmed SN, Siddiqi ZA (2006). Antiepileptic drugs and liver disease. Seizure.

[55] Litwin AH, Soloway I, Gourevitch MN (2005). Integrating services for injection drug users infected with hepatitis C virus with methadone maintenance treatment: challenges and opportunities. Clinical Infect Dis.

[56] Sylvestre DL, Zweben JE (2007). Integrating HCV services for drug users: a model to improve engagement and outcomes. Int J Drug Policy.

[57] Proeschold-Bell RJ, Patkar AA, Naggie S, Coward L, Mannelli P, Yao J (2012). An integrated alcohol abuse and medical treatment model for patients with hepatitis C. Dig Dis Sci.

